# Pilot study of humanized glypican-3-targeted zirconium-89 immuno-positron emission tomography for hepatocellular carcinoma

**DOI:** 10.1186/s13550-024-01134-1

**Published:** 2024-08-22

**Authors:** Lindsay K. Dickerson, Adrienne L. Lehnert, Donald K. Hamlin, Kevin P. Labadie, Kristin E. Goodsell, Yongjun Liu, Yawen Li, D. Scott Wilbur, Robert Miyaoka, James O. Park

**Affiliations:** 1 Department of Surgery, University of Washington, 1959 NE Pacific St., Box 356410, Seattle, WA 98195 USA; 2 Department of Radiology, University of Washington, Seattle, WA USA; 3 Department of Radiation Oncology, University of Washington, Seattle, WA USA; 4 Department of Laboratory Medicine and Pathology, University of Washington, Seattle, WA USA; 5 Department of Surgery, Icahn School of Medicine at Mount Sinai, New York, USA

## Background

Hepatocellular carcinoma (HCC) is increasing in incidence worldwide and has become the fastest growing cause of cancer death in the United States, with a median survival of less than 1 year [1–3]. In order to improve survival with current treatments, HCC must be detected early when it is amenable to surgical resection or transplantation [3, 4]. However, multiphase, computed tomography (CT) or magnetic resonance imaging frequently misses lesions less than 1 cm, resulting in diagnostic uncertainty, delayed diagnosis, and early recurrence following resection [5–7]. Innovative technology capable of detecting HCC with enhanced sensitivity and specificity is therefore imperative and pressing.

Radioisotope theranostics, including immuno-positron emission tomography (immunoPET) and radioimmunotherapy (RIT), is an emerging field with the potential to transform HCC diagnosis and therapy [8]. While yttrium-90 microspheres and iodine-131-labeled lipiodol and metuximab are used in radioembolization therapy, there are currently no FDA approved theranostics for HCC [9]. However, glypican-3 (GPC3)-targeted radioisotopes have shown promise in preclinical and early clinical studies [6, 9–20]. GPC3 is a cell surface antigen expressed on up to 80% of HCCs but absent in liver parenchyma and benign lesions, making it an accessible and specific target for a theranostic approach [14, 15, 21]. GPC3-based imaging has the potential to facilitate earlier, definitive HCC diagnosis and subsequent RIT, thus improving patient survival [16].

Our group previously demonstrated that immunoPET using ^89^Zr-labeled murine antibody targeting GPC3 (^89^Zr-αGPC3_M_) reliably identified small HCCs in mice [6, 10, 11]. Natarajan et al. described the use of ^89^Zr-labeled humanized αGPC3 for HCC detection in a patient-derived xenograft model [16]. We built on this important work by humanizing our radioimmunoconjugate (αGPC3_H_) and performing in vitro and novel in vivo comparisons to its murine predecessor. Here, we report that ^89^Zr-αGPC3_H_ targets GPC3 comparably to ^89^Zr-αGPC3_M_, resulting in highly specific tumor uptake and successful HCC detection.

## Methods

Creative Biolabs, Inc. (Shirley, NY) constructed αGPC3_H_ by engrafting of the parental murine antibody’s complementarity-determining region (CDR). Flow cytometry was used to evaluate in vitro binding of αGPC3_M_, a chimeric intermediary (αGPC3_C_), αGPC3_H_, and αGPC3-deferoxamine (DFO) to HepG2 cells. Specifically, HepG2 cells were resuspended in cold FACS buffer and aliquoted at a concentration of 1 × 10^6^ cells/100 μL. Primary and secondary antibodies were sequentially added to the cell suspensions and incubated at 4 °C protected from light for 30 min each. Cells were then washed, resuspended in cold buffer, and analyzed with the Symphony A3 (BD Biosciences, San Jose, CA) flow cytometer. A minimum of 10,000 cells were analyzed for each sample. Data analysis was performed using FlowJo software (TreeStar, Ashland, OR).

Orthotopic xenograft models of HCC were generated as previously described in athymic nude mice (Jackson Laboratories) [10–12, 22]. A final concentration of 1 × 10^6^ HepG2 cells in 20 μL Geltrex (Gibco, Billings, MT) was injected into the left hepatic lobe. Two weeks after injection, bioluminescence imaging (BLI) was performed using the In Vivo Imaging System Spectrum (PerkinElmer) to verify tumor establishment. Tumor size was calculated in a semi-quantitative manner based on mean photon emission (photons/sec) in a 2D region of interest (ROI) containing the tumor.

Humanized and murine αGPC3 were conjugated with eight equivalents of deferoxamine (DFO)-N-chlorosuccinimide and labeled with positron emitter ^89^Zr as previously described [10]. Isoelectric focusing was consistent with an average of less than one DFO moiety per antibody in both groups. The radiochemical yield of ^89^Zr-DFO-αGPC3_H_ and ^89^Zr-DFO-αGPC3_M_ was 98% and 89%, respectively. Radiochemical purity was determined by instant thin layer chromatography (iTLC) and confirmed by radioactive HPLC. Specific activity was calculated by dividing total radioactivity (GBq) of ^89^Zr by total mg for each antibody. For simplicity, ^89^Zr-DFO-αGPC3 is written as ^89^Zr-αGPC3.

Mice were injected retro-orbitally with 8.1 to 10 megabecquerels (MBq) of ^89^Zr-αGPC3_H_ (n = 11) or ^89^Zr-αGPC3_M_ (n = 11). Five days after injection, mice with tumors predicted using BLI (n = 6 per group) underwent imaging using the Inveon small-animal PET/CT scanner (Siemens Medical Solutions USA, Inc. Molecular Imaging, Knoxville, TN). Horos software (Nimble Co., Annapolis, MD) was used for image analysis. Maximum activity concentration (MBq/mL) was measured in a 2D region of interest (ROI) to calculate tumor radioisotope uptake (percent injected dose per milliliter, %ID/mL), tumor-to-liver ratio, and tumor maximum standardized uptake value (SUV_max_).

Biodistribution studies were performed separately in non-tumor-bearing, non-imaged mice 2 days after injection and in PET-imaged mice after imaging completion. Ionization events (counts per minute, CPM) were measured for each organ and tumor (when applicable) using a Cobra II gamma counter (Packard Bioscience, Meriden, CT) and tissue uptake (%ID/g) was calculated. Livers from PET-imaged mice were processed for histopathology. Details provided in Supplementary Methods.

## Results

### *Humanized αGPC3 and αGPC3-DFO maintains GPC3 binding *in vitro

**Fig. 1:**
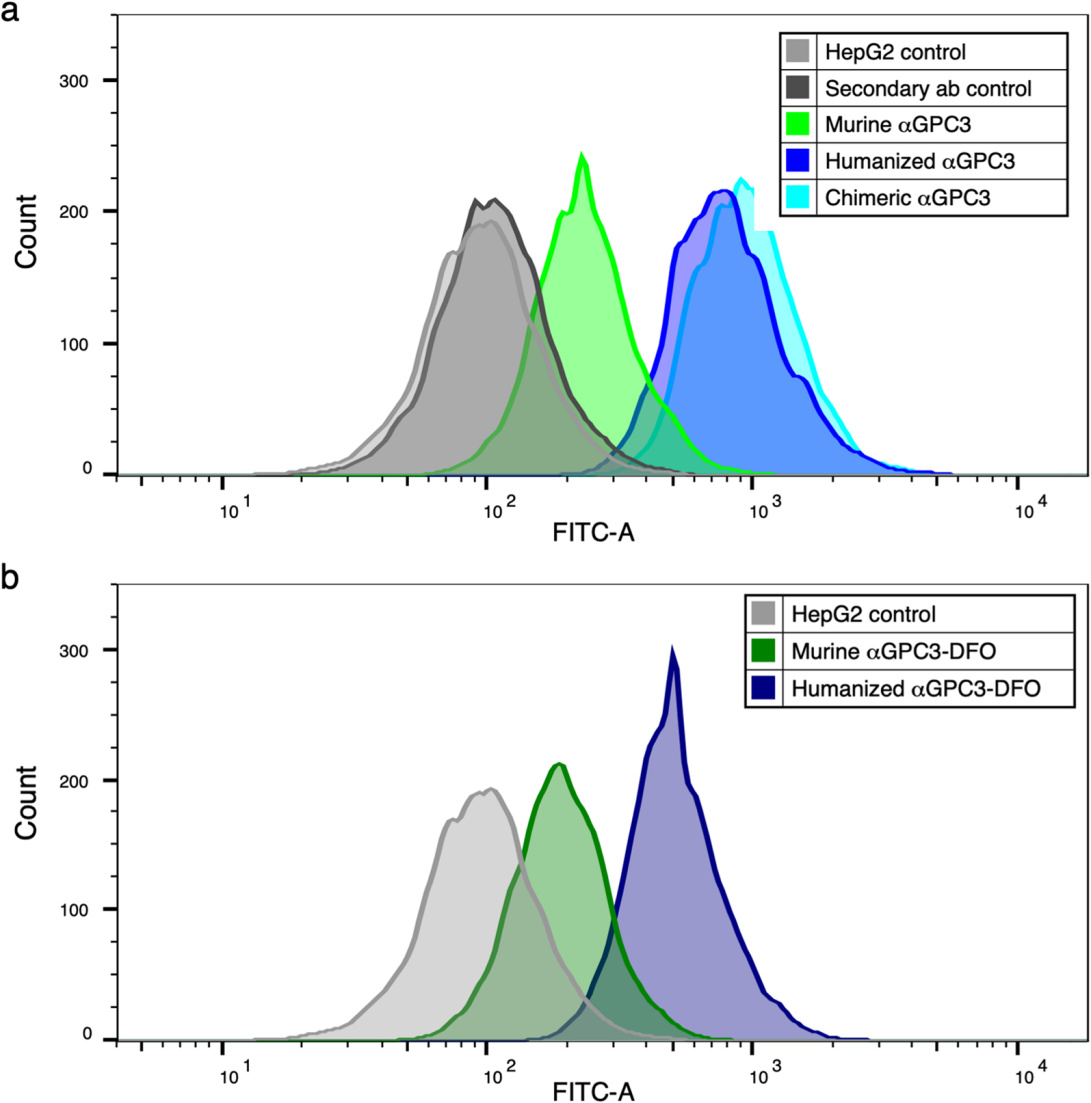
Humanized αGPC3 binds to GPC3-expressing HepG2 cells in vitro. **a** Flow cytometric mean fluorescence intensity (MFI) of αGPC3_M_ (green), αGPC3_C_ (aqua), and αGPC3_H_ (blue) compared with controls (unlabeled HepG2 cells and FITC-labeled secondary antibody alone, gray). **b** MFI of deferoxamine (DFO)-conjugated αGPC3_M_ (dark green) and αGPC3_H_ (navy) compared with control (gray)

Binding to HepG2 cell surface GPC3 by unconjugated αGPC3_M_, αGPC3_C_, and αGPC3_H_ was confirmed by flow cytometry (Fig. 1a). Binding of DFO-conjugated and αGPC3_M_ to GPC3 was overall similar to the unconjugated antibody (Fig. 1b). Binding of αGPC3_H_ and αGPC3_C_ to GPC3 was greater than αGPC3_M_.

### Bioluminescence imaging predicts tumor establishment

**Fig. 2:**
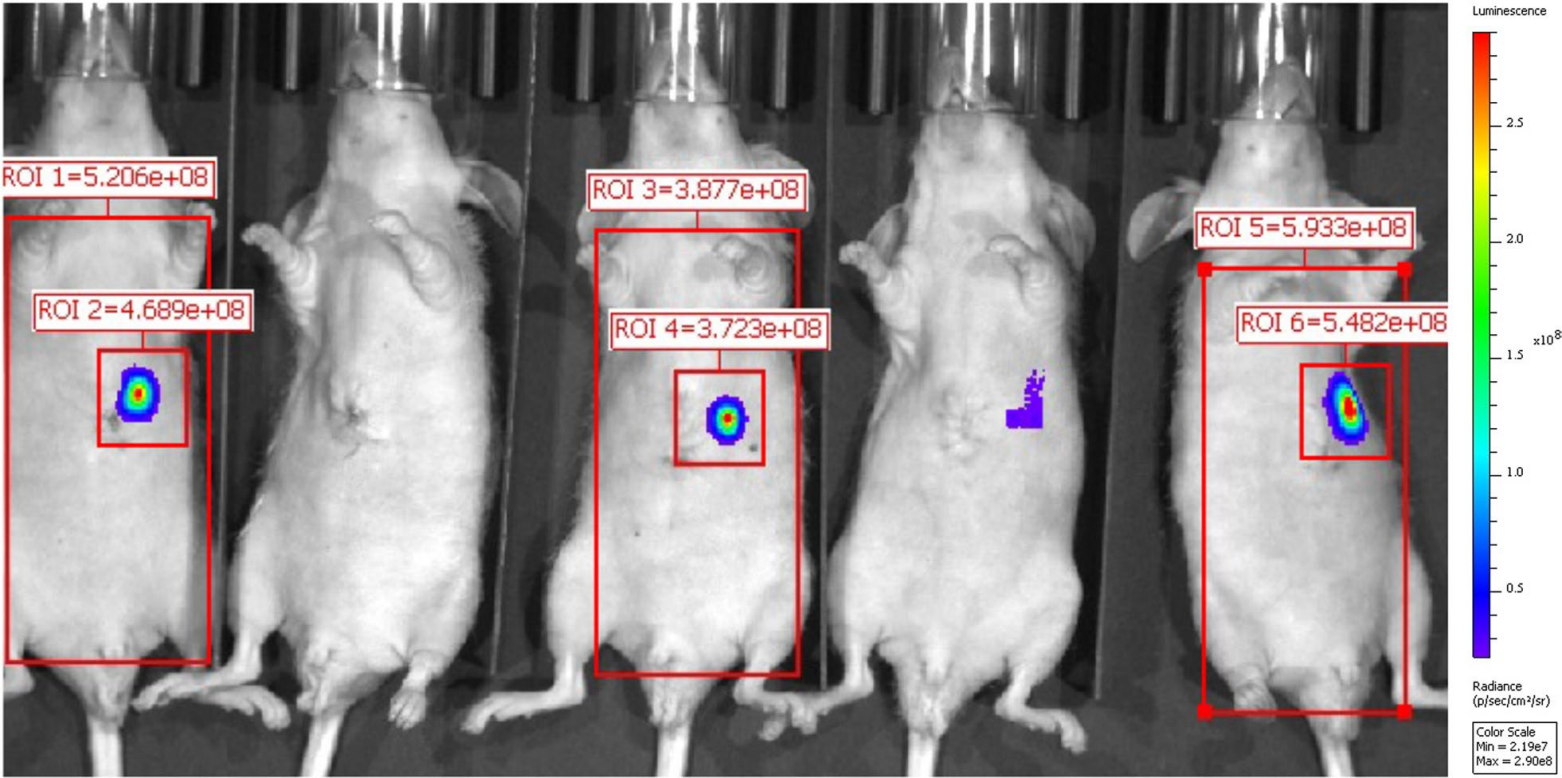
Bioluminescence imaging predicts tumor establishment. Representative BLI image demonstrating predicted tumor establishment in three of the five mice shown. ROI = region of interest. (Position 1–5 from left to right; mean photon emission not calculated for positions two and four)

Tumors were identified with BLI (Fig. 2). Mice were assigned to ^89^Zr-αGPC3_H_ and ^89^Zr-αGPC3_M_ injection such that mean photon emission (photons/sec) in tumor-containing ROIs was similar between groups (4.1 × 10^8^ vs. 5.3 × 10^8^ photons/s; Table 1).

**Table 1:** ImmunoPET and biodistribution data by mouse

	H1	H2	H3	H4	H5	H6^b^	M7	M8	M9	M10	M11	M12^b^
Bioluminescence imaging												
Biolum (photons/ second)	3.7 × 10^8^	5.5 × 10^8^	a	9.2 × 10^8^	7 × 10^7^	1.6 × 10^8^	1.4 × 10^8^	4.1 × 10^8^	2.5 × 10^8^	2.1 × 10^9^	4.7 × 10^8^	2 × 10^7^
PET/CT imaging												
Tumor uptake (%ID/mL)	24	131	67	245	21		15.5	14.9	24	142	111	
Liver uptake (%ID/mL)	12.5	7.9	9.6	7.3	8.1	8.9	6.7	7.0	5.4	5.9	5.6	4.7
Tumor- liver ratio	1.9	16.5	6.9	34	2.6		2.3	2.1	4.5	24	19.9	
SUV_max_	9.2	47	23	104	9.1		6.0	5.6	9.0	55	45	
Biodistribution studies												
Tumor uptake (%ID/g)	− 426^c^	100	982	109	85		16.8	58	458	156	56	
Liver uptake (%ID/g)	20	10.2	16.2	11.3	10.5	10.9	7.6	8.3	6.1	6.4	6.5	7.7
Tumor- liver ratio	− 21^c^	9.8	61	9.6	8.1		2.2	7.0	75	24	8.8	
Histopathologic analysis												
Tumor weight (g)	0.003	0.031	0.005	0.115	0.003		0.003	0.003	0.003	0.036	0.043	

^a^Not measured due to minimal apparent signal

^b^H6, M12: no tumor identified on PET/CT or histopathology (blank = not applicable)

^c^PET and histologic findings suggest a tumor was present in H1, and the negative %ID/g was due to standard experimental error and a calculated baseline liver uptake that was higher than the measured tumor-containing left hepatic lobe uptake

### αGPC3_H_ is amenable to ^89^Zr radiolabeling

The radiochemical purity of both ^89^Zr-αGPC3 antibodies was > 98% and the specific activity was 0.14 GBq/mg. Details provided in Supplementary Methods.

### Humanized ^89^Zr-αGPC3 immunoPET reliably identifies tumors

**Fig. 3:**
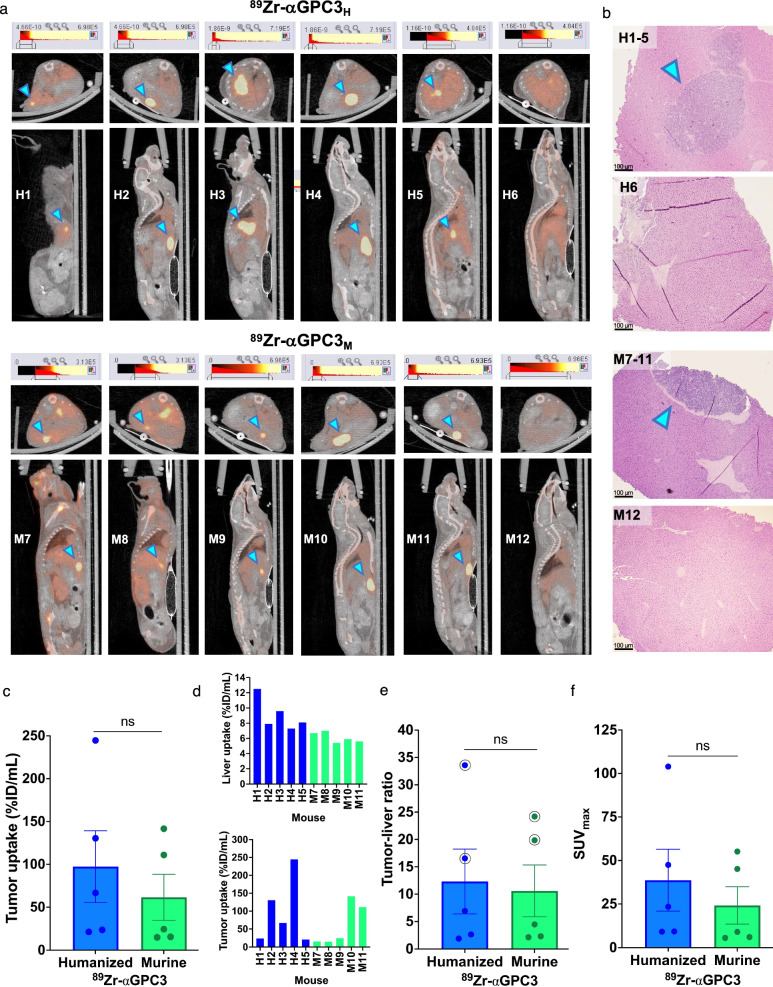
Humanized ^89^Zr-αGPC3 immunoPET reliably identifies tumors. **a** Axial (top) and sagittal (bottom) PET/CT images of ^89^Zr-αGPC3_H_- and ^89^Zr-αGPC3_M_-injected mice. **b** Select H&E-stained liver sections. Blue arrowheads denote tumors. Scale bars 100 μm. **c** Tumor radioisotope uptake (%ID/mL). **d** Liver and tumor %ID/mL by mouse. **e** Tumor-to-liver ratio of %ID/mL; circle denotes largest tumors. **f** Tumor maximum standardized uptake (SUV_max_). Each point or thin bar (**d**) denotes the value for each tumor-bearing mouse (n = 5/group); large bars denote the mean with SEM

Five of six mice injected with ^89^Zr-αGPC3_H_ and ^89^Zr-αGPC3_M_, respectively, demonstrated increased PET intensity within a focal area in the liver consistent with a tumor (mice H1–H5 and M7–M11; Fig. 3a). Mean bioluminescence calculated from prior IVIS measurements (Table 1) was equivalent between groups (4.8 × 10^8^ vs. 6.3 × 10^8^ ± 4.5 × 10^8^ photon/sec, *p* = 0.75). Histopathologic analysis identified tumors in mice H1-H5 and M7-M11, but not in mice without PET-identified tumors (H6, M12) (Fig. 3b). Tumor uptake (97 vs. 61 ± 50%ID/mL, *p* = 0.42) and tumor-to-liver ratios (12 vs. 11 ± 7.6, *p* = 0.68) were not significantly different between groups, despite significantly increased liver uptake in the ^89^Zr-αGPC3_H_-injected mice (9.1 vs 6.1 ± 1.0%ID/mL, *p* = 0.02) (Fig. 3c–e). SUV_max_ was equivalent between groups (39 vs. 24 ± 21, *p* = 0.51) (Fig. 3f).

### No difference in tumor radioimmunoconjugate uptake on biodistribution studies

**Fig. 4:**
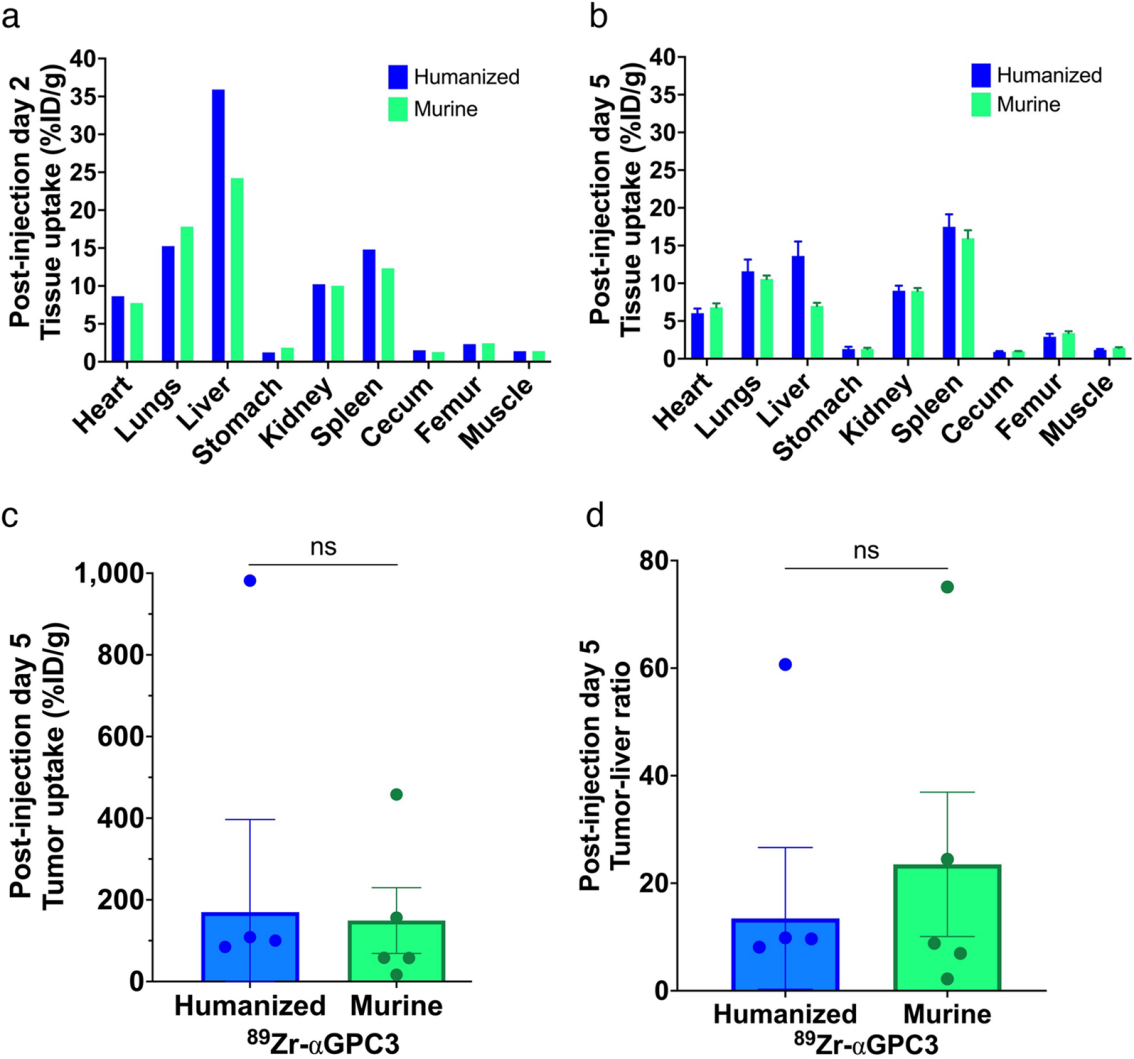
Tissue biodistribution is not significantly different between humanized and murine ^89^Zr-αGPC3-injected mice. **a**, **b** Mean tissue radioisotope uptake (%ID/g) from biodistribution studies in (**a**) mice not imaged with PET (non-tumor-bearing) (n = 5/group) and **b** imaged with PET (majority tumor-bearing) (n = 6/group), 2 and 5 days after injection, respectively. *Note* For mice not imaged with PET (**a**), organ uptake was calculated as an injection group mean, and thus error bars are not included. **c** Tumor uptake and **d** tumor-to-liver ratio (%ID/g) from biodistribution studies in PET-imaged mice. Each point denotes the value for each tumor-bearing mouse (humanized n = 5, murine n = 5; negative values from mouse H1 were included in mean tumor uptake and tumor-to-liver ratio calculations for Fig. 4c, d, but the points are not shown on graph). Bars denote mean with SEM when applicable

In non-imaged mice, the liver had the highest %ID/g calculated from gamma counter measurements followed by the lungs and spleen, with no significant difference between ^89^Zr-αGPC3_H_ and ^89^Zr-αGPC3_M_-injected mice (mean 10 vs. 8.8 ± 4.6%ID/g, *p* = 0.77) (Fig. 4a). In PET-imaged mice, tumor uptake was sevenfold greater than other organs, with equivalent organ uptake (mean 7.1 vs. 6.3 ± 2.7%ID/g, *p* = 0.75), tumor uptake (170 vs. 149 ± 241%ID/g, *p* = 0.93), and tumor-to-liver ratio of %ID/g (13 vs 24 ± 19, *p* = 0.61) (Fig. 4b–d).

## Discussion

Humanized αGPC3 specifically targeted GPC3 in vitro and in vivo*,* enabling HCC detection with immunoPET in an orthotopic xenograft mouse model. This proof-of-concept study builds on our prior research validating a murine radioimmunoconjugate for a theranostic approach to HCC, with potential to improve diagnosis, treatment, and survival [6, 10–12].

Our results demonstrate that humanization of ^89^Zr-αGPC3 did not alter the highly avid binding to GPC3 on HepG2 cells and liver tumor xenografts. First, flow cytometry established at least equivalent, if not greater, binding of αGPC3_H_ to GPC3 compared with αGPC3_M_, with minimal change when conjugated with DFO. Next, quality assurance of ^89^Zr labeling confirmed that ^89^Zr-αGPC3_H_ maintained high purity and specific activity. The majority of our experiments focused on the novel in vivo comparison between ^89^Zr-αGPC3_H_ and ^89^Zr-αGPC3_M_*.* Five of six tumors in each group were detected by immunoPET, with no difference between groups in mean bioluminescence. PET/CT data revealed no significant difference in mean tumor uptake and tumor-to-liver ratios (%ID/mL). Similarly, biodistribution analysis showed no difference in mean organ uptake, tumor uptake, and tumor-to-liver ratios (%ID/g).

While finding comparability between ^89^Zr-αGPC3_H_ and ^89^Zr-αGPC3_M_ achieved the study’s primary goal, additional details are worth noting. First, tumor uptake varied based on tumor size, with higher uptake in larger tumors as previously demonstrated [6]. %ID/g (gamma counter) results were greater than %ID/mL (PET) due to limited PET/CT spatial resolution causing partial volume effect; hence, there could be a larger discrepancy between %ID/g and %ID/mL values in mice with smaller tumors (e.g., H3, M9) (Fig. 3, Table 1). While further consideration of the clinical impact of partial volume effect is warranted, this finding does highlight the successful detection of a range of tumor sizes with ^89^Zr-αGPC3 immunoPET. Second, background liver uptake was greater in the ^89^Zr-αGPC3_H_ group, which could imply Fc-mediated liver uptake of ^89^Zr-αGPC3_H_. However, our prior studies of mice injected with ^89^Zr-αGPC3_M_ compared with non-GPC3-targeting and GPC3-blocked controls demonstrated similar background liver uptake [6, 11, 16]. Furthermore, the tumor-to-liver ratio by nature adjusts for such variables, with no difference between groups suggesting that tumor uptake was also proportionally higher in the humanized antibody group. In fact, tumor-to-liver ratios of 12 or greater indicate ^89^Zr-αGPC3_H_ is highly specific for GPC3-expressing tumors [16]. Finally, a limitation of our study is that we did not measure blood pool activity in biodistribution analyses and thus could not calculate tumor-to-blood ratios. However, we did include other highly vascular organs, and have shown in prior studies that ^89^Zr-αGPC3 uptake in tumor and organs such as the liver, kidneys, and spleen is greater than blood pool activity beyond 24 h after injection [6, 11].

Tumor presence was histopathologically confirmed in mice with PET-identified tumors, while no tumors were found on histologic analysis of livers without PET-identified tumors. A second limitation of our study is that, while meticulous gross examination of the liver and histopathologic analysis of suspected tumors was performed, serial sectioning of the entire left hepatic lobe was not undertaken due to funding constraints. Therefore, the discordance between BLI and PET for H6 and M12 is unresolved. Of note, the 3-week interval between imaging modalities was longer than in previous studies and thus tumor involution may have occurred.

Our study is similar to those from other groups in that it underscores the potential of human αGPC3 to detect HCC with immunoPET, however, there are key differences. Tumor-to-liver ratios by PET/CT and biodistribution analyses were notably higher than those reported by Natarajan et al. using a similar ^89^Zr-labeled human αGPC3 IgG antibody and Fayn et al. using ^89^Zr-labeled GPC3-targeting HN3 single-domain antibodies. In addition, there was a greater relative difference between tumor uptake and uptake in organs such as the heart, lungs, gastrointestinal tract, and kidneys on biodistribution analysis [16, 17]. While different methods for model development and radioimmunoconjugate injection used may affect the results such that they are not directly comparable [16, 17], it is possible that our humanized antibody has a higher specificity for GPC3-expressing tumors. Furthermore, it should be noted that tumor-to-liver ratios were measured 5 days after injection in this study compared with 1–7 days after injection in the aforementioned studies, however our prior experiments with ^89^Zr-αGPC3_M_ demonstrated high tumor-to-liver ratios calculated from 4 h up to 7 days after injection [6, 11]. Finally, Carrasquillo et al. conducted a phase I clinical study of PET/CT in HCC patients using αGPC3 codrituzumab labeled with iodine-124 (^124^I). While this valuable work underscores the clinical translatability of radiolabeled antibodies against GPC3, there was no tumor uptake in one patient and low tumor-to-liver ratios in several others [18]. The authors stated that ^89^Zr could have been a reasonable alternative to ^124^I, and our findings support further investigation of ^89^Zr-αGPC3 immunoPET to overcome challenges encountered with other radioimmunoconjugates. We appreciate the rigorous and ongoing work by our colleagues in the field and believe that parallel approaches to developing GPC3-targeted radiolabeled imaging agents will be beneficial [14–20, 23, 24].

## Conclusions

Humanized αGPC3 successfully targeted GPC3 in vitro and in vivo. Compared with our previously validated murine antibody, ^89^Zr-αGPC3_H_ immunoPET demonstrated comparable HCC detection with highly specific tumor uptake in an orthotopic xenograft mouse model, affirming the efficacy and clinical translatability of ^89^Zr-αGPC3_H_ immunoPET for HCC detection [16]. Given our GPC3-targeted murine radioimmunoconjugates were previously validated for both immunoPET and cytotoxic RIT, immediate next steps include assessing treatment response using αGPC3_H_-based RIT. This developing theranostic joins a growing field of other solid tumors, including colorectal, breast, prostate, renal cell cancers, non-Hodgkin’s lymphoma, and neuroendocrine tumors, and has the potential to transform HCC management [10, 25, 26].

### Supplementary Information

Supplementary Material 1.

## Data Availability

All data analyzed during the current study are available from the corresponding author on reasonable request.

## Abbreviations

GPC3: Glypican-3
immunoPET: Immuno-positron emission tomography
^89^Zr-αGPC3: Zirconium-89-labeled anti-GPC3 antibody
HCC: Hepatocellular carcinoma
CT: Computed tomography
RIT: Radioimmunotherapy
CDR: Complementarity-determining region
DFO: Deferoxamine
BLI: Bioluminescence imaging
MBq: Megabecquerels
ROI: Region of interest
%ID/mL: Percent injected dose per milliliter
SUV_max_: Maximum standardized uptake value
^124^I: Iodine-124

## References

[1] Philips CA, Rajesh S, Nair DC, Ahamed R, Abduljaleel JK, Augustine P. Hepatocellular carcinoma in 2021: an exhaustive update. Cureus. 2021. 10.7759/cureus.19274. 34754704 10.7759/cureus.19274 PMC8569837

[2] Chen ZY, Chhatwal J. Changing epidemiology of hepatocellular carcinoma and role of surveillance. In: Hoshida Y, editor. Hepatocellular carcinoma: translational precision medicine approaches. Totowa: Humana Press; 2019. 32078270

[3] Singal AG, Parikh ND, Rich NE, John BV, Pillai A. Hepatocellular carcinoma surveillance and staging. In: Hoshida Y, editor. Hepatocellular carcinoma: translational precision medicine approaches. Totowa: Humana Press; 2019. 32078269

[4] Llovet JM, Kelley RK, Villanueva A, et al. Hepatocellular carcinoma. Nat Rev Dis Primers. 2021. 10.1038/s41572-020-00240-3. 33479224 10.1038/s41572-020-00240-3 PMC13537433

[5] Ayyappan AP, Jhaveri KS. CT and MRI of hepatocellular carcinoma: an update. Expert Rev Anticancer Ther. 2010. 10.1586/era.10.24. 20397916 10.1586/era.10.24

[6] Sham JG, Kievit FM, Grierson JR, et al. Gypican-3-targeted ^89^Zr PET imaging of hepatocellular carcinoma. J Nuc Med. 2014. 10.2967/jnumed.113.132118. 10.2967/jnumed.113.132118 PMC4116087 24627434

[7] Choi J-Y, Lee J-M, Sirlin CB. CT and MR imaging diagnosis and staging of hepatocellular carcinoma: part II. Extracellular agents, hepatobiliary agents, and ancillary imaging features. Radiology. 2014;273(1):30–50. 10.1148/radiol.14132362. 25247563 10.1148/radiol.14132362 PMC4263770

[8] Jacobson O, Chen X. Interrogating tumor metabolism and tumor microenvironments using molecular positron emission tomography imaging. Theranostic approaches to improve therapeutics. Pharmacol Rev. 2013;65(4):1214–56. 10.1124/pr.113.007625. 24064460 10.1124/pr.113.007625 PMC3799232

[9] Chen H, Teng M, Zhang H, Liang X, Cheng H, Liu G. Advanced radionuclides in diagnosis and therapy for hepatocellular carcinoma. Chin Chem Lett. 2022. 10.1016/j.cclet.2022.03.079.36091579 10.1016/j.cclet.2022.03.079

[10] Labadie KP, Ludwig AD, Lehnert AL, et al. Glypican-3 targeted delivery of ^89^Zr and ^90^Y as a theranostic radionuclide platform for hepatocellular carcinoma. Sci Rep. 2021. 10.1038/s41598-021-82172-w. 33580090 10.1038/s41598-021-82172-w PMC7881163

[11] Labadie KP, Lehnert AL, Kenoyer AL, et al. Glypican-3 targeted positron emission tomography detects sub-centimeter tumors in a xenograft model of hepatocellular carcinoma. Eur J Nucl Med Mol Imaging Res. 2023. 10.1186/s13550-023-00980-9. 10.1186/s13550-023-00980-9 PMC10140215 37103671

[12] Ludwig AD, Labadie KP, Seo YD, et al. Yttrium-90-labeled anti-glypican 3 radioimmunotherapy halts tumor growth in an orthotopic xenograft model of hepatocellular carcinoma. J Oncol. 2019. 10.1155/2019/4564707. 31636665 10.1155/2019/4564707 PMC6766125

[13] Yang X, Liu H, Sun CK, et al. Imaging of hepatocellular carcinoma patient-derived xenografts using ^89^Zr-labeled anti-glypican-3 monoclonal antibody. Biomaterials. 2014. 10.1016/j.biomaterials.2014.04.089. 24836949 10.1016/j.biomaterials.2014.04.089 PMC4363564

[14] Nishida T, Kataoka H. Glypican 3-targeted therapy in hepatocellular carcinoma. Cancers. 2019. 10.3390/cancers11091339. 31510063 10.3390/cancers11091339 PMC6770328

[15] Grega SD, Zheng DX, Zheng Q-H. Imaging ligands targeting glypican-3 receptor expression in hepatocellular carcinoma. Am J Nucl Med Mol Imaging. 2022;12(4):113–21. 36072763 PMC9441927

[16] Natarajan A, Zhang H, Ye W, et al. A humanized anti-GPC3 antibody for immuno-positron emission tomography imaging of orthotopic mouse model of patient-derived hepatocellular carcinoma xenografts. Cancers. 2021. 10.3390/cancers13163977. 34439132 10.3390/cancers13163977 PMC8391944

[17] Fayn S, King AP, Gutsche NT, et al. Site-specifically conjugated single-domain antibody successfully identifies glypican-3-expressing liver cancer by immuno-PET. J Nuc Med. 2023. 10.2967/jnumed.122.265171. 10.2967/jnumed.122.265171 PMC10315705 36997331

[18] Carrasquillo JA, O’Donoghue JA, Beylergil V, et al. I-124 codrituzumab imaging and biodistribution in patients with hepatocellular carcinoma. Eur J Nucl Med Mol Imaging Res. 2018. 10.1186/s13550-018-0374-8. 10.1186/s13550-018-0374-8 PMC5838028 29508107

[19] Hanaoka H, Nagaya T, Sato K, et al. Glypican-3 targeted human heavy chain antibody as a drug carrier for hepatocellular carcinoma therapy. Mol Pharm. 2015. 10.1021/acs.mocpharmaceut.5b00132. 25955255 10.1021/acs.mocpharmaceut.5b00132 PMC7720675

[20] An S, Zhang D, Zhang Y, et al. GPC3-targeted immunoPET imaging of hepatocellular carcinomas. Eur J Nucl Med Mol Imaging. 2022. 10.1007/s00259-022-05723-x. 35147737 10.1007/s00259-022-05723-x

[21] Wang HL, Anatelli F, Zhai QJ, Adley B, Chuang S-T, Yang XJ. Glypican-3 as a useful diagnostic marker that distinguishes hepatocellular carcinoma from benign hepatocellular mass lesions. Arch Pathol Lab Med. 2008. 10.5858/132.11.1723. 18976006 10.5858/132.11.1723

[22] Perciedusert N, Hurst V, Ahluwalia A, et al. The ARRIVE guidelines 2.0: updated guidelines for reporting animal research. PLoS Biol. 2020;18(7):e3000410. 10.1371/journal.pbio.3000410. 32663219 10.1371/journal.pbio.3000410 PMC7360023

[23] Lin F, Clift R, Ehara T, et al. Peptide binder to glypican-3 as a theranostic agent for hepatocellular carcinoma. J Nucl Med. 2024. 10.2967/jnumed.123.266766. 38423788 10.2967/jnumed.123.266766

[24] Bal C, Ballal S, Kallur K, et al. Abstract 2585: first in human study with a novel peptide binder to glypican-3, demonstrates high specificity as a PET imaging agent in patients with hepatocellular carcinoma. Cancer Res. 2024. 10.1158/1538-7445.AM2024-2585. 10.1158/1538-7445.AM2024-2585

[25] Labadie KP, Hamlin DK, Kenoyer A, et al. Glypican-3-targeted ^227^Th α-therapy reduces tumor burden in an orthotopic xenograft murine model of hepatocellular carcinoma. J Nucl Med. 2022. 10.2967/jnumed.121.262562. 34772791 10.2967/jnumed.121.262562 PMC9258570

[26] Werner RA, Weich A, Kricher M, et al. The theranostic promise for neuroendocrine tumors in the late 2010s—Where do we stand, where do we go? Theranostics. 2018;8(22):6088. 10.7150/thno.30357. 30613284 10.7150/thno.30357 PMC6299695

