# Supplementary material for: Pilot study of humanized glypican-3-targeted zirconium-89 immuno-positron emission tomography for hepatocellular carcinoma
Source: EJNMMI Res. 2024 Aug 22;14:74. doi: 10.1186/s13550-024-01134-1 (PMC11341507; doi:10.1186/s13550-024-01134-1)
Supplement: Supplementary file 1 — Supplementary Material 1. [file 13550_2024_1134_MOESM1_ESM.docx]

**Title:** Pilot study of humanized glypican-3-targeted zirconium-89 immuno-positron emission tomography for hepatocellular carcinoma

**Authors:** Lindsay K. Dickerson, Adrienne L. Lehnert, Donald K. Hamlin, Kevin P. Labadie, Kristin E. Goodsell, Yongjun Liu, Yawen Li, D. Scott Wilbur, Robert Miyaoka, James O. Park^*^

^*^Department of Surgery, University of Washington, Seattle, WA, USA; jopark@uw.edu

**Journal**: EJNMMI

**Supplementary Methods**

*Cell lines*

HepG2-Red-Fluc (HepG2) cells which express *Luciferase* and high levels of GPC3 (RRID:CVCL_5I98, Bioware BW134280, PerkinElmer, Waltham, MA) were established and maintained as previously described [10-12]. Cells were cultured in a monolayer at 37°C in Dulbecco’s Modified Eagle Medium (DMEM; Gibco, Thermo Fisher Scientific, Waltham, MA) with 4.5 g/L glucose, L-glutamine, and sodium pyruvate supplemented with 10% HyClone characterized fetal bovine serum (FBS; Thermo Fisher Scientific) in a humidified chamber with 5% CO_2_. Cells were grown until 70-80% confluent and passaged per manufacturer’s instructions (PerkinElmer).

*Production of murine* α*GPC3*

Murine immunoglobulin G1 (IgG1) αGPC3 antibody (αGPC3_M_)-producing hybridomas were generated through the Fred Hutchinson Cancer Center antibody core facility as previously described [6].

*Production of chimeric and humanized* α*GPC3*

Creative Biolabs, Inc. (Shirley, NY) developed a chimeric αGPC3 antibody (αGPC3_C_) consisting of a humanized Fc region and a murine antigen binding fragment region of IgG1. After flow cytometric confirmation of *in vitro* binding of αGPC3_C_, Creative Biolabs subsequently developed a fully humanized αGPC3 (αGPC3_H_) antibody. This entailed computational modeling of the variable regions and a search for the most appropriate human heavy and light chain variable region framework acceptors. After the in silico engrafting of the murine antibody's Complementarity-Determining Region (which recognizes the target antigen) into human IgG1 using an authenticated human antibody scaffold grafting technique, putative back mutations in the acceptor framework were designed, resulting in three heavy chain variable and three light chain variable candidate sequences for a total of nine humanization clones. The International Immunogenetics Information System DomainGapAlign tool was used to analyze the humanization percentage of all the heavy chain variable and light chain variable candidate sequences. By using one candidate sequence with the highest humanization percentage (huVH1v1VLv1) as representative of the humanized antibody designs, an immunogenicity assessment was performed, including T cell epitope, B cell epitope, and MHC II epitope studies. The predicted antigenicity epitopes for the VHv1 sequence were GSELKKPGA, KVSCK, APGQ, SVS, IS, and AE and for the VLv1 sequence were LGQP, RPGQSPRR, and GSGTDF. All predicted epitopes contained backmutations, which were retained. Following in silico post-translational modifications and aggregation assessments of the parental murine antibody and huVH1v1VLv1, expression and binding ability verification evaluations were completed for the top five humanized candidates. The top-performing candidate was selected and production was expanded. Characteristics of the chosen αGPC3_H_ antibody: EC_50_: 0.51 ng/mL, determined by ELISA; purity (monomer percentage): 99%, determined by size exclusion-high performance liquid chromatography (HPLC); amino acid sequence heavy chain: QVQLVQSGSELKKPGASVKVSCK

ASGYTFTDYSMHWVRQAPGQGLEWMGWINTETGESTYADDFKGRFVFSLTSVSTAYLQISSLKAEDTAVYYCAAPVWGAGTTVTVSS*;* amino acid sequence light chain*:* DVVMTQSPLSLSVTLGQP

ASISCKSSQSLLYTNGKTYLNWLQQRPGQSPRRLIYLVSKLDSGVPDRFSGSGSGTDFLKISRVEAEDVGVYYCLQGTHFPRTFGGGTKVEIK.

*Flow cytometry*

HepG2 cells were resuspended in cold FACS buffer (1X PBS with 2% FBS and 0.5 mM EDTA (Invitrogen, Thermo Fisher Scientific)) and aliquoted to Eppendorf tubes at a concentration of 1x10^6^ cells/100 μL. 5 μg/mL of primary antibodies (αGPC3_M_, αGPC3_M_-DFO, αGPC3_C_, αGPC3_H_, and αGPC3_H_-DFO) were added to the cell suspensions and incubated at 4°C protected from light for 30 minutes. After incubation, samples were washed in cold buffer and incubated with 4 μg/mL of FITC-labeled mouse anti-human IgG Fc secondary antibody (Invitrogen MA110379, Thermo Fisher Scientific, conc. 0.1 mg/mL) or FITC-labeled mouse IgGκ secondary antibody (Santa Cruz Biotechnology sc516140, conc. 200 μg/0.5 mL) at 4°C protected from light for 30 min.

*Development of orthotopic HCC xenograft model*

The orthotopic xenograft model was generated as previously described in 8-week-old athymic nude mice (Nu/J, Jackson Laboratories, Bar Harbor, ME) [10-12,22]. After more than one week of acclimatizing in the animal facility, mice were anesthetized using 2% inhaled isoflurane and the left hepatic lobe was exposed through a 0.7 cm upper midline laparotomy. HepG2 cells had been detached, suspended in DMEM with FBS, and pelleted via centrifugation. The cell pellet was resuspended in 400 μL Geltrex (Gibco, Billings, MT) to achieve a final concentration of 2x10^7^ cells. 20 μL of the Geltrex-HepG2 suspension (1x10^6^ cells) was slowly injected into the subcapsular space of the left hepatic lobe using a 100 μL syringe with 25-gauge needle (Restek, Thermo Fisher Scientific). The laparotomy was closed in two layers as previously described [11] and standard postoperative care was performed.

*Bioluminescence imaging*

12 days after hepatic subcapsular cell injection, mice were anesthetized using 2% inhaled isoflurane,100 mg/kg intraperitoneal injection of VivoGlo Luciferin (Promega, Madison, WI) was administered, mice were placed on a warmed stage, and bioluminescence imaging (BLI) was performed using the In Vivo Imaging System Spectrum (PerkinElmer) to verify tumor establishment. Mice were assigned to ^89^Zr-αGPC3_H_ and ^89^Zr-αGPC3_M_ injection such that bioluminescence in the tumor-containing ROIs was similar between groups (Table 1).

*Small-animal positron emission tomography*

One month after hepatic subcapsular HepG2 cell injection, 22 mice were injected retro-orbitally with humanized (n=11) and murine (n=11) ^89^Zr-αGPC3. Each 200 μL injection contained 70 μg of antibody, or 2.8x10^14^ molecules (0.47 nanomoles). The total injected dose measured by a dose calibrator (starting dose minus residual dose) ranged from 8.1 to 10 megabecquerels (MBq) (219 to 271 uCi). The mean injected dose per group +/- SD was 8.7 +/- 0.47 MBq for ^89^Zr-αGPC3_H_ and 9.7 +/- 0.42 MBq for ^89^Zr-αGPC3_M_.

^89^Zr-αGPC3 imaging studies were performed using the Inveon small-animal positron emission/computed tomography (PET/CT) scanner (Siemens Medical Solutions USA, Inc. Molecular Imaging, Knoxville, TN), which was calibrated for ^89^Zr. On day five after ^89^Zr-αGPC3 injection, mice with tumors visualized on BLI (humanized n=6, murine n=6) were anesthetized with 2% inhaled isoflurane in 100% oxygen at 1L/min and imaged on a temperature-controlled bed with respiratory monitoring. Mice had a 30 min PET scan followed by a 15 min CT scan, which enabled scatter and attenuation correction. PET images were reconstructed using ordered subset expectation maximization/shifted Poisson maximum a posteriori (OSEM3D/SP-MAP; 2 iterations 18 subsets) with a 256×256 matrix, target resolution of 1.5mm, zoom factor 1.3, and corrections for scatter and attenuation. CT images were reconstructed using forward back projection with a Shepp-Logan filter, slight noise reduction, and appropriate beam hardening corrections.

*Positron emission/computed tomography analysis*

Horos software (Nimble Co., Annapolis, MD) was used for image analysis. Tumor and liver activity concentration (MBq/mL) was determined by creating a uniform 2D ROI within the tumor and non-tumor-containing right hepatic lobe, respectively. Tumor ROIs avoided tumor boundaries in order to minimize partial volume effect. Because of the relatively large correction factors needed to compensate for partial volume and the non-spherical shape of the tumors, we chose not to apply any correction as it might significantly increase the variability in the measurements, thus not necessarily improving the interpretability of our results. Tumor and liver radioisotope uptake (percent injected dose of radioisotope per mL (%ID/mL)) was calculated for each mouse by dividing maximum activity concentration by the decay-corrected total injected radioisotope dose (averaged for each group), comparing mean %ID/mL between ^89^Zr-αGPC3_M_ and ^89^Zr-αGPC3_H_-injected mice. Tumor-to-liver ratio of %ID/mL was determined for each mouse, comparing the mean between groups. Tumor maximum standardized uptake (SUV_max_) was calculated by dividing maximum activity concentration (MBq/mL) in the tumor ROI by the total mouse activity per mouse mass (MBq/g), decay corrected to the time of imaging.

*Biodistribution studies*

Organs were harvested for tissue biodistribution in mice not imaged with PET (non-tumor-bearing) two days after ^89^Zr-αGPC3 injection (humanized n=5, murine n=5). Organs including tumor-bearing livers were harvested from PET-imaged mice after imaging was completed, or five days after injection (humanized n=6, murine n=6). This methodology allowed for two biodistribution timepoints. Mice were euthanized, perfused with isotonic saline, and organs including heart, lungs, tumor-bearing liver (left hepatic lobe), non-tumor-bearing liver (right hepatic lobe), stomach, kidney, spleen, cecum, right femur, and right leg muscle were harvested and weighed. For mice not imaged with PET (non-tumor-bearing), only the right hepatic lobe was harvested. Ionization events (counts per minute, CPM) were measured for each organ using a Cobra II gamma counter (Packard Bioscience, Meriden, CT). Tumor CPM was determined by subtracting weight-adjusted baseline liver CPM from the measured CPM of the tumor-containing left hepatic lobe. The tissue uptake (%ID/g) for tumors and organs was calculated by dividing decay-corrected CPM by specimen weight, converting this to MBq/g, and dividing by total injected radioisotope dose (averaged for each group). Organ uptake was calculated for individual PET-imaged mice and as an injection group mean for mice not imaged with PET (Fig. 4a-b). Tumor and liver %ID/g were calculated for each PET-imaged mouse, and these values were used to determine the tumor-to-liver ratios of absorbed activity in %ID/g. Negative values for H1 were included in mean tumor uptake and tumor-to-liver ratio calculations (Fig. 4c-d; points not shown on graph). As noted in the Table 1 legend, PET and histologic findings suggest a tumor was present in H1, and the negative %ID/g was due to standard experimental error and a calculated baseline liver uptake that was higher than the measured tumor-containing left hepatic lobe uptake. In fact, liver uptake in H1 was the highest of the cohort in both PET and biodistribution analyses (Table 1).

*Histopathologic analysis*

After biodistribution analysis, tumor- and non-tumor-bearing livers (left lobe) from PET-imaged animals were fixed in 10% phosphate-buffered formalin for four weeks. Meticulous gross examination of the liver was performed to assess for the presence of tumors (LKD, JOP). Suspected tumors were dissected from the livers, weighed for biodistribution %ID/g calculations, embedded in paraffin, and sectioned onto positively charged slides. Slide groups included: H1-H5 (^89^Zr-αGPC3_H_-injected, PET-identified), H6 (^89^Zr-αGPC3_H_-injected, not identified on PET), M7-M11 (^89^Zr-αGPC3_M_-injected, PET-identified), M12 (^89^Zr-αGPC3_M_-injected, not identified on PET). Hematoxylin and eosin staining was performed on all slides, which were reviewed by an expert pathologist (YL) for the presence of tumors. While meticulous gross dissection of livers and serial sectioning of suspected tumors were performed, serial sectioning of the entire left hepatic lobe where HepG2 cells were injected [10] to definitively confirm or refute the presence of a tumor in H6 and M12 was not undertaken due to financial constraints.

*Statistical analysis*

All numeric and graphical data are expressed as mean +/- SEM unless otherwise indicated. Prism GraphPad was used for statistical analysis. Unpaired, two-tailed Student’s t-tests were used, with a p-value < 0.05 considered statistically significant.
